# Functional outcome after late cranioplasty after decompressive craniectomy: a single-center retrospective study

**DOI:** 10.1007/s00068-024-02479-x

**Published:** 2024-03-01

**Authors:** Tim Lampmann, Harun Asoglu, Johannes Weller, Anna-Laura Potthoff, Matthias Schneider, Mohammed Banat, Frank Alexander Schildberg, Hartmut Vatter, Motaz Hamed, Valeri Borger

**Affiliations:** 1 Department of Neurosurgery, University Hospital Bonn, Venusberg Campus 1, 53127 Bonn, Germany; 2 Department of Neurology, University Hospital Bonn, Venusberg Campus 1, 53127 Bonn, Germany; 3 Department of Orthopaedics and Trauma Surgery, University Hospital Bonn, Venusberg Campus 1, 53127 Bonn, Germany

**Keywords:** Cranioplasty, Outcome, Decompressive craniectomy, Subarachnoid hemorrhage, Stroke, Traumatic brain injury, Intracerebral hemorrhage

## Abstract

**Objective:**

The best time for cranioplasty (CP) after decompressive craniectomy (DC) is controversial, and there are no authoritative guidelines yet. Both complications as well as outcome may depend on the timing of CP. The aim of this single-center study was to evaluate the impact of late CP on procedural safety as well as on patient outcome.

**Methods:**

All patients receiving CP at a tertiary university medical center between 01/2015 and 12/2022 were included retrospectively. Patients’ conditions were assessed according to the modified Rankin Scale (mRS) prior to CP and 6 months after. Baseline characteristics, indication for DC, time from DC to CP, and postoperative complications according to the Landriel Ibañez Classification were analyzed.

**Results:**

CP was performed in 271 patients who previously underwent DC due to traumatic brain injury (25.5%), ischemic stroke (29.5%), aneurysmal subarachnoid hemorrhage (26.9%), or intracerebral hemorrhage (18.1%). The median interval between DC and CP was 143 days (interquartile range 112–184 days). Receiver operating characteristic analysis revealed a cut-off of 149 days, where CP performed within 149 days after DC led to an improvement on mRS after CP (*p* = 0.001). In multivariate analysis, additional rehabilitation after and better mRS before CP were independently associated with improvement of outcome. The rate of complications was similar between early and late CP (24.8% and 25.4%, respectively, *p* = 0.562).

**Conclusions:**

Late cranioplasty is a safe procedure. The outcome was improved when additional rehabilitation was performed after cranioplasty and was not associated with the timing of cranioplasty.

## Introduction

Decompressive craniectomy (DC) is a life-saving procedure in specific neurological diseases associated with refractory intracranial pressure elevation including traumatic brain injury (TBI), ischemic stroke (IS), aneurysmal subarachnoid hemorrhage (SAH), and intracerebral hemorrhage (ICH) [1–4]. Patients that survived the acute phase may attend rehabilitation and desire subsequent cranioplasty (CP) afterwards. Besides the obvious benefits in terms of neurocranial protection and cosmetic improvement, CP may also improve neurological recovery [5, 6]. However, CP carries complications itself and therefore may influence outcome [7, 8]. The best time-point for CP is controversially discussed in literature [3, 9]. Complications as well as outcome may depend on the timing of CP [9, 10]. There are some prospective studies that mostly focus on complications of CP, but do not recommend timing of CP in matters of functional outcome [7, 8, 11, 12].

At the authors’ institution, CP is mostly performed between 3 to 6 months after DC, but was sometimes delayed, especially during the COVID-19 pandemic. The aim of this single-center study was to evaluate the safety of later CP and its influence on functional outcome.

## Materials and methods

### Study design and patient characteristics

A total of 356 consecutive adult (≥ 18 years) patients received autologous or allogeneic CP at the authors’ institution between 01/2015 and 12/2022. We retrospectively collected baseline characteristics in those patients including age, sex, side of DC, cause of DC, time from DC to CP, complications within 30 days after CP according to the Landriel Ibañez Classification [13], clinical condition assessed by the modified Rankin Scale (mRS) prior to CP and 6 months after CP, and admission to rehabilitation after CP. We included all cases with the following indications for DC in further analyses: TBI, IS, SAH, and ICH. Infectious diseases and allogenic CP due to aseptic bone-flap resorption were excluded.

### Clinical management

Autologous bone flaps were stored under sterile conditions in cryopreservation at − 80 °C. CP was mostly performed 3 months after DC as described previously [6, 9]. Before CP, any anticoagulation or antiplatelet drugs were withdrawn. We evaluated the inflammation parameters (e.g., C-reactive protein or procalcitonine) at admission prior to CP. If inflammation parameters were elevated and an infectious disease was assumed, CP was postponed. When secondary neurological deterioration and morphological sunken flap occurred (sinking-skin-flap syndrome), CP would have been performed as timely as possible. Allogenic CP was performed when the bone flap was either defective due to the initial TBI or when DC has been performed at another center. All patients received a perioperative infection prophylaxis. A postoperative computed tomography was performed immediately after surgery to evaluate positioning of CP and exclude early complications. Considering the patients’ condition and will, a subsequent rehabilitation after CP was initiated. Rehabilitation was defined as inpatient or outpatient rehabilitation performed at a specialized neurorehabilitative institution after discharge.

### Statistical analysis

Data were analyzed using SPSS Statistics (Version 27, IBM Corp. Armonk, NY, USA). Outcome was calculated as “ΔmRS” = “mRS before CP” – “mRS at 6 months after CP,” where ΔmRS > 0 indicates that the patients’ condition improved, ΔmRS = 0 indicates that the patients’ condition was unchanged, and ΔmRS < 0 indicates that the patients’ condition deteriorated.

Continuous data were expressed as median with interquartile range (IQR), and categorical variables were expressed as absolute and relative frequencies. Mann–Whitney U test was used to test for differences of ordinal data. Categorical variables were analyzed in contingency tables using the two-sided Pearson’s *Χ*^2^ test.

Receiver-operating characteristic (ROC) analysis was performed to evaluate how the time from DC to CP was associated with outcome, dichotomized in improvement (ΔmRS > 0) vs. no improvement (ΔmRS ≤ 0), and with complications according to the Landriel Ibañez Classification, dichotomized in no complications vs. any complication (grade I–IV). The Youden index was used to select the best cut-off point. *P*-values < 0.05 were considered statistically significant.

A backward stepwise method was used to construct a multivariable logistic regression model in relation to an improved outcome after CP (ΔmRS > 0) as a dependent variable with an inclusion criterion of *P*-value < 0.05.

## Results

**Fig. 1:**
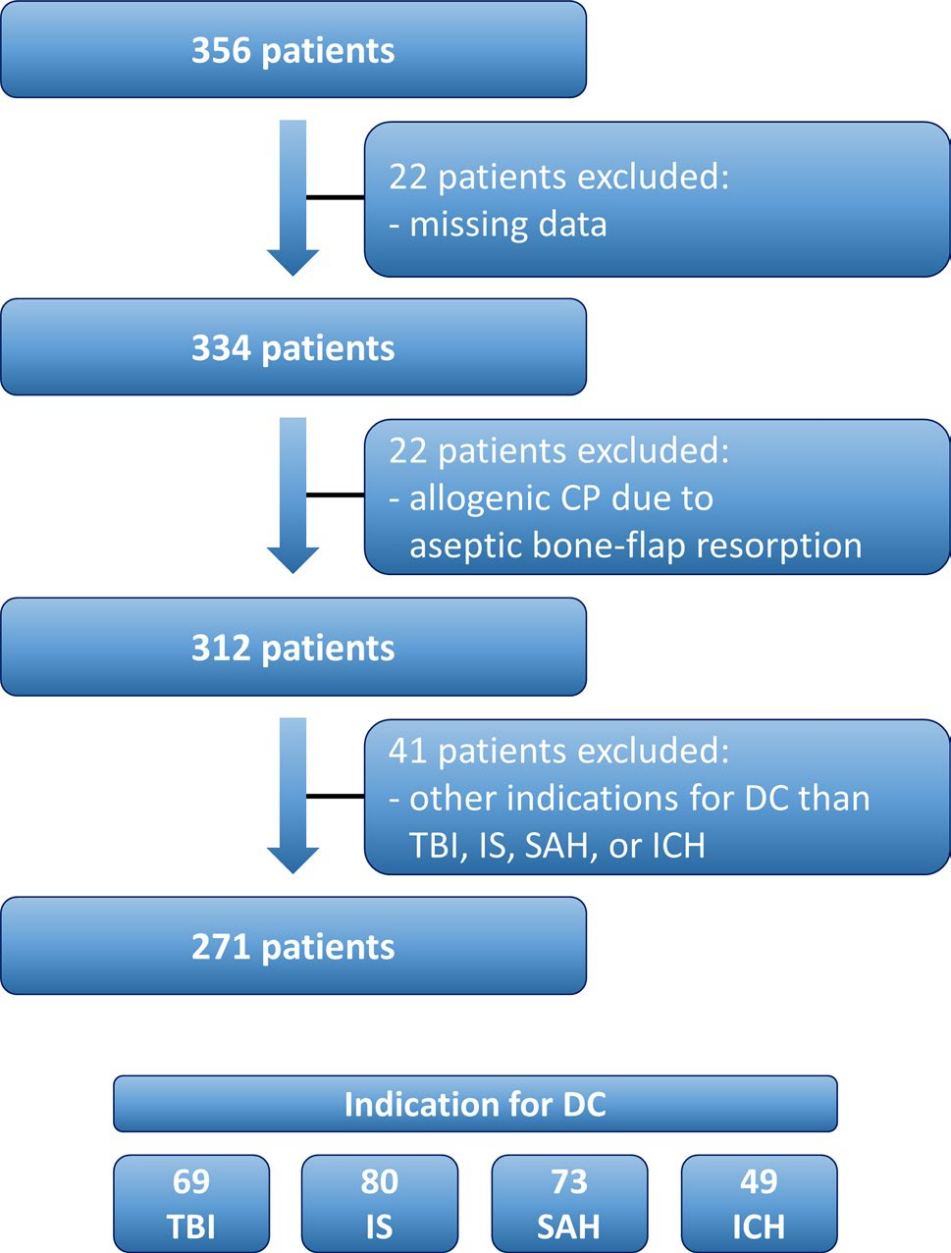
Flow chart illustrating the selection process of consecutive CP patients. CP, cranioplasty; DC, decompressive craniectomy; ICH, intracerebral hemorrhage; IS, ischemic stroke; SAH, subarachnoid hemorrhage; TBI, traumatic brain injury

From 356 patients receiving CP between 01/2015 and 12/2022 at the authors’ institution, 271 fulfilled the inclusion criteria and were included in this study (Fig. 1). The median age was 54 years, and 45% were female. The median time from DC to CP was 143 days with a total range of 72–726 days. DC was more frequently performed on the right side, and indications (TBI, IS, SAH, and ICH) showed similar frequencies. However, no case of sinking-skin-flap syndrome was observed in the current study. Further patient characteristics are shown in Table 1.

**Table 1:** Patient characteristics

	***N*** (%)
Overall	271 (100%)
Median age at DC in years (IQR)	54 (44.2–61.9)
Sex	
Female	122 (45.0%)
Male	149 (55.0%)
Side	
Right	151 (55.7%)
Left	112 (41.3%)
Bilateral	8 (3.0%)
Indication for DC	
TBI	69 (25.5%)
IS	80 (29.5%)
SAH	73 (26.9%)
ICH	49 (18.1%)
Median time to CP in days (IQR)	143 (112–184)
Timeframe of CP	
About 3 months (± 14 days)	40 (14.8%)
Between 3 and 6 months	160 (59.0%)
After 6 months	71 (26.2%)
Material	
Autologous	260 (95.9%)
Allogenic	11 (4.1%)
Landriel Ibañez Classification	
No complication	203 (74.9%)
Grade I a	23 (8.5%)
Grade I b	20 (7.4%)
Grade II a	5 (1.8%)
Grade II b	5 (1.8%)
Grade III a	8 (3.0%)
Grade III b	6 (2.2%)
Grade IV	1 (0.4%)
Permanent CSF shunt	
Before CP	76 (28.0%)
After CP	11 (4.1%)
Median mRS before CP (IQR)	4 (4–5)
Rehabilitation after CP	
Yes	156 (57.6%)
No	115 (42.4%)
Median mRS 6 months after CP (IQR)	4 (3–5)
Outcome 6 months after CP	
Improved (ΔmRS > 0)	79 (29.2%)
Unchanged (ΔmRS = 0)	187 (69.0%)
Deteriorated (ΔmRS < 0)	5 (1.8%)

*CP*, cranioplasty; *CSF*, cerebrospinal fluid; *DC*, decompressive craniectomy; *ICH*, intracerebral hemorrhage; *IQR*, interquartile range; *IS*, ischemic stroke; *mRS*, modified Rankin Scale; *SAH*, subarachnoid hemorrhage; *TBI*, traumatic brain injury

**Fig. 2:**
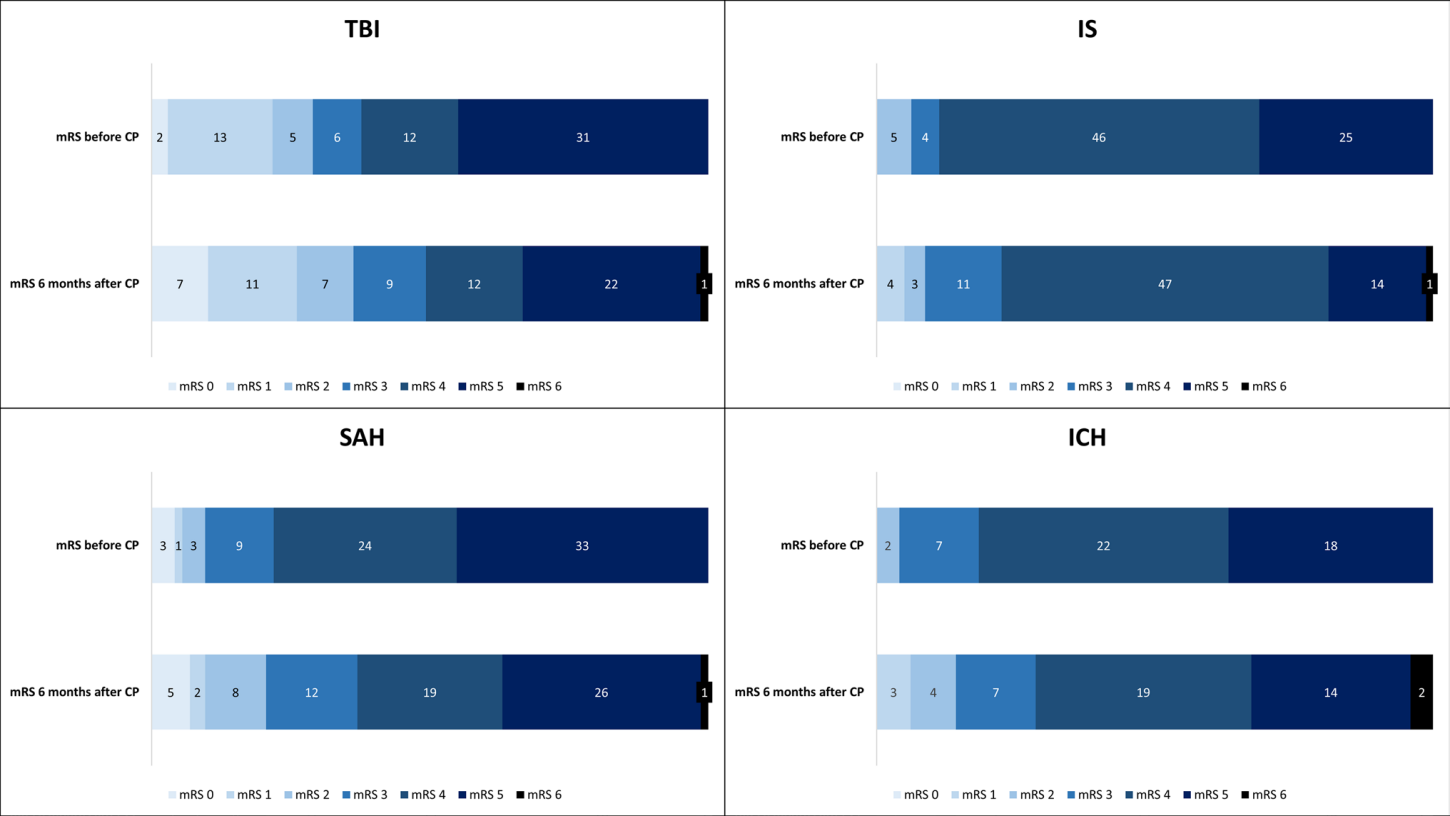
Grotta bars illustrating the mRS scores before and after CP according to the indication for decompressive craniectomy. CP, cranioplasty; ICH, intracerebral hemorrhage; IS, ischemic stroke; mRS, modified Rankin Scale; SAH, subarachnoid hemorrhage; TBI, traumatic brain injury

The different mRS scores before CP and 6 months after CP regarding the indication for DC are visualized in Fig. 2.

**Fig. 3:**
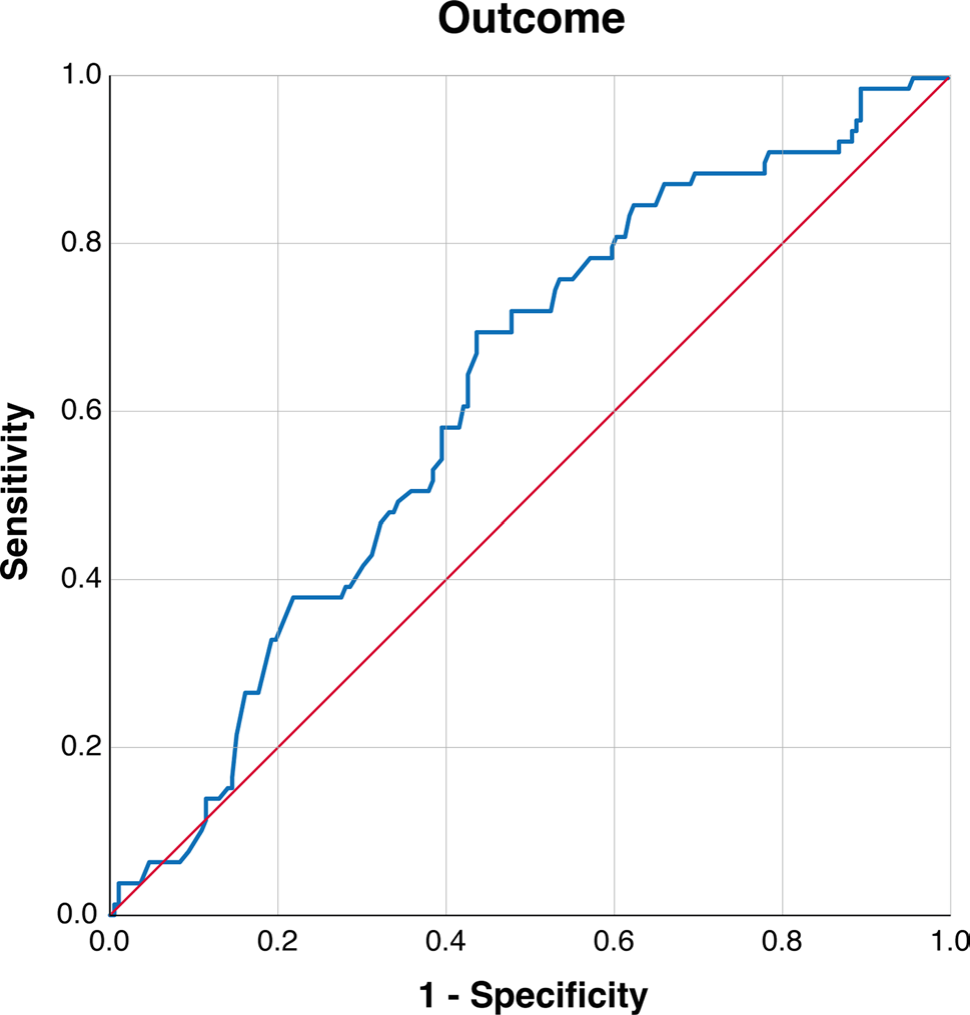
Receiver-operating characteristic curve illustrating time to cranioplasty in prediction of outcome 6 months after CP

ROC curves were constructed to investigate the association between time to CP and changes in 6-month outcome and procedural complications. The AUC of time to CP for the prediction of outcome improvement at 6 months after CP (ΔmRS ≤ 0 vs. ΔmRS > 0) was 0.62 (95% CI: 0.55–0.69; *p* = 0.002). Sensitivity and specificity of the time to CP threshold of 149 days (according to the Youden index) were 72.2% and 52.1%, respectively. Figure 3 shows the ROC curve of time to CP predicting the outcome.

**Fig. 4:**
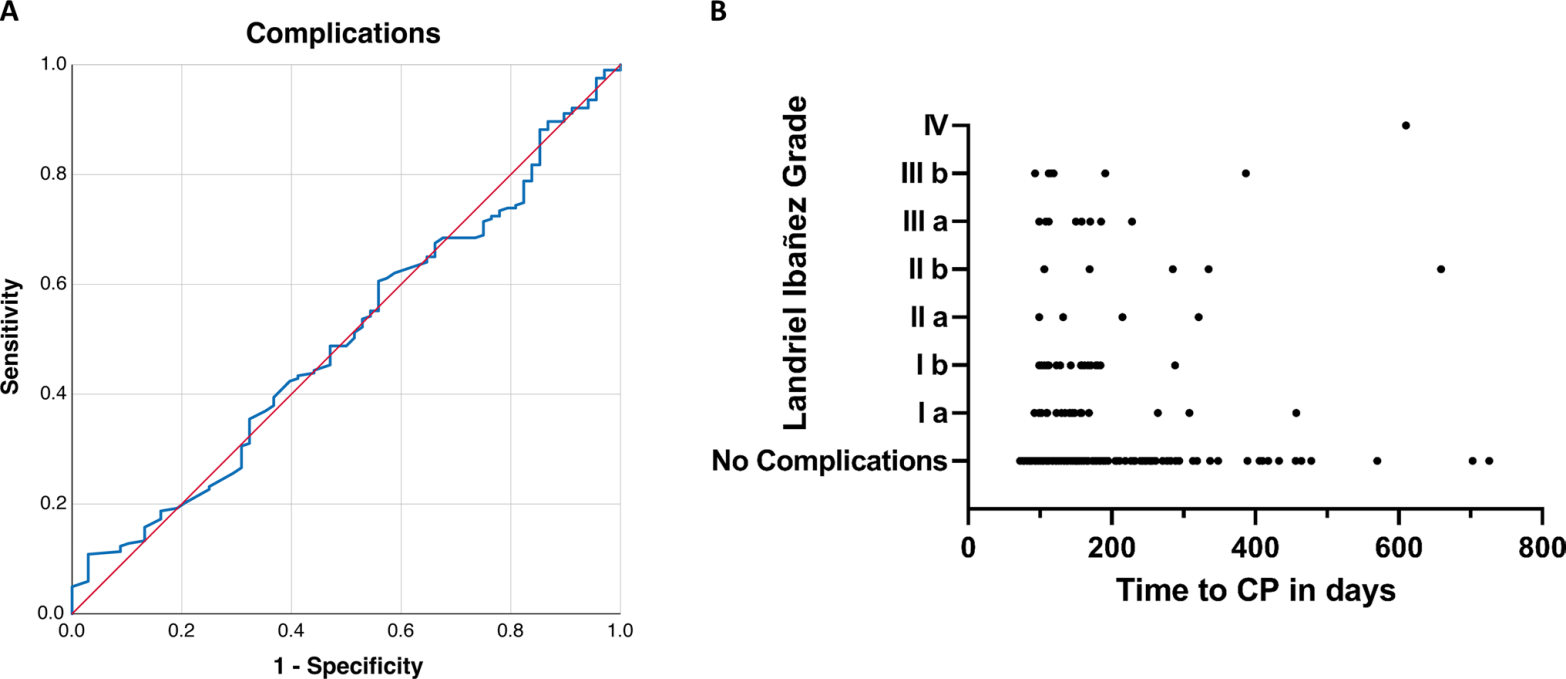
**A** Receiver-operating characteristic curve illustrating time to cranioplasty in prediction of complications (Landriel Ibañez Grade I–IV) 30 days after CP. **B** Scatter plot demonstrating the relation of complication grade according to the Landriel Ibañez Classification to time to CP in days

In contrast to these findings, the time to CP was not associated with procedural complications. The AUC of time to CP for complications after CP was 0.50 (95% CI: 0.42–0.58; *p* = 0.98). Figure 2 shows the ROC curve of time to CP predicting complications after CP and the temporal distribution of complications depending on the time to CP. Using the cut-off from ROC analysis for outcome improvement, the patients could be dichotomized into an early (≤ 149 days) and late (> 149 days) group of time from DC to CP. There was no difference in the rate of complications between those groups or overall in ROC analysis (Fig. 4).

Further comparison of early vs. late CP groups revealed that more patients attended rehabilitation after CP, when CP was performed within 149 days (69.8% vs. 42.6%, *p* < 0.001). Comparing clinical outcomes according to the underlying pathology leading to craniectomy, the median mRS before and after CP was higher in patients undergoing late CP (*p* = 0.019 and *p* < 0.001, respectively), which was mainly driven by ICH patients (*p* = 0.018 and *p* = 0.002, respectively). Table 2 summarizes the results of the analysis between the both groups, early and late CP.

**Table 2:** Comparison of early (≤ 149 days) vs. late (> 149 days) cranioplasty

	Early CP (≤ 149 days)	Late CP (> 149 days)	*P*-value
Overall	149	122	
Median age at DC in years (IQR)	53.3 (41.1–61.2)	54.6 (45.4–63.0)	0.251
Sex			1.000
Female	67 (45.0%)	55 (45.1%)	
Male	82 (55.0%)	67 (54.9%)	
Side			0.183
Right	81 (54.4%)	70 (57.4%)	
Left	61 (40.9%)	51 (41.8%)	
Bilateral	7 (4.7%)	1 (0.8%)	
Indication for DC			0.115
TBI	45 (30.2%)	24 (19.7%)	
IS	43 (28.9%)	37 (30.3%)	
SAH	40 (26.8%)	33 (27.0%)	
ICH	21 (14.1%)	28 (23.0%)	
Median time to CP in days (IQR)	115 (103–129)	205 (164–283)	NA
Landriel Ibañez Classification			0.562
No complication	112 (75.2%)	91 (74.6%)	
Grade I a	15 (10.1%)	8 (6.6%)	
Grade I b	11 (7.4%)	9 (7.4%)	
Grade II a	3 (2.0%)	2 (1.6%)	
Grade II b	1 (0.7%)	4 (3.3%)	
Grade III a	3 (2.0%)	5 (4.1%)	
Grade III b	4 (2.7%)	2 (1.6%)	
Grade IV	0 (0%)	1 (0.8%)	
Permanent CSF shunt			0.089
Yes	41 (27.5%)	46 (37.7%)	
No	108 (72.5%)	76 (62.3%)	
Median mRS before CP (IQR)	4 (3–5)	4 (4–5)	**0.019**
TBI	4 (1–5)	4.5 (3–5)	0.410
IS	4 (4–5)	4 (4–5)	0.827
SAH	4 (3–5)	5 (4–5)	0.136
ICH	4 (3–4)	4.5 (4–5)	**0.018**
Median mRS 6 months after CP (IQR)	4 (2–4)	4 (4–5)	**< 0.001**
TBI	3 (1–5)	4 (2–5)	0.209
IS	4 (3–4)	4 (4–4.5)	0.105
SAH	4 (2–5)	4 (3–5)	0.233
ICH	4 (2–4)	4 (4–5)	**0.002**
Rehabilitation after CP			**< 0.001**
Yes	104 (69.8%)	52 (42.6%)	
No	45 (30.2%)	70 (57.4%)	
Outcome 6 months after CP			**0.001**
Improved (ΔmRS > 0)	57 (38.3%)	22 (18.0%)	
Unchanged (ΔmRS = 0)	90 (60.4%)	97 (79.5%)	
Deteriorated (ΔmRS < 0)	2 (1.3%)	3 (2.5%)	
Improved outcome 6 months after CP			
TBI	17 (37.8%)	4 (16.7%)	0.053
IS	18 (41.9%)	6 (16.2%)	**0.010**
SAH	14 (35.0%)	9 (27.3%)	0.530
ICH	8 (38.1%)	3 (10.7%)	**0.027**

*CP*, cranioplasty; *CSF*, cerebrospinal fluid; *DC*, decompressive craniectomy; *ICH*, intracerebral hemorrhage; *IQR*, interquartile range; *IS*, ischemic stroke; *mRS*, modified Rankin Scale; *SAH*, subarachnoid hemorrhage; *TBI*, traumatic brain injury. Significant *P*-values < 0.05 are marked bold

As more patients attended rehabilitation in the early CP group (69.8% vs. 42.6%, *p* < 0.001), we further compared patients with vs. without rehabilitation after CP. There was no difference in age, sex, or indication for CP between both groups. When patients attended rehabilitation after CP, outcome 6 months after CP improved more frequently (38.5% vs. 16.5%, *p* < 0.001). Table 3 summarizes the results of the analysis between the both groups, rehabilitation and no rehabilitation after CP.

**Table 3:** Comparison of patients undergoing rehabilitation vs. no rehabilitation after cranioplasty

	Rehabilitation	No rehabilitation	*P*-value
Overall	156	115	
Median age at DC in years (IQR)	53.8 (42.5–61.4)	54.1 (45.8–63.0)	0.549
Sex			0.622
Female	68 (43.6%)	54 (47.0%)	
Male	88 (56.4%)	61 (53.0%)	
Side			0.344
Right	82 (52.6%)	69 (60.0%)	
Left	70 (44.9%)	42 (36.5%)	
Bilateral	4 (2.6%)	4 (3.5%)	
Indication for DC			0.824
TBI	38 (24.4%)	31 (27.0%)	
IS	49 (31.4%)	31 (27.0%)	
SAH	40 (25.6%)	33 (28.7%)	
ICH	29 (18.6%)	20 (17.3%)	
Median time to CP in days (IQR)	132 (110–161)	159 (116–256)	**< 0.001**
Landriel Ibañez Classification			0.511
No complication	114 (73.1%)	89 (77.4%)	
Grade I a	15 (9.6%)	8 (7.0%)	
Grade I b	14 (9.0%)	6 (5.2%)	
Grade II a	3 (1.9%)	2 (1.7%)	
Grade II b	1 (0.6%)	4 (3.5%)	
Grade III a	5 (3.2%)	3 (2.6%)	
Grade III b	4 (2.6%)	2 (1.7%)	
Grade IV	0 (0%)	1 (0.9%)	
Median mRS before CP (IQR)	4 (4–5)	4 (3–5)	**< 0.001**
Median mRS 6 months after CP (IQR)	4 (3–5)	4 (2–5)	0.292
Outcome 6 months after CP			**< 0.001**
Improved (ΔmRS > 0)	60 (38.5%)	19 (16.5%)	
Unchanged (ΔmRS = 0)	95 (60.9%)	92 (80.0%)	
Deteriorated (ΔmRS < 0)	1 (0.6%)	4 (3.5%)	

*CP*, cranioplasty; *DC*, decompressive craniectomy; *ICH*, intracerebral hemorrhage; *IQR*, interquartile range; *IS*, ischemic stroke; *mRS*, modified Rankin Scale; *SAH*, subarachnoid hemorrhage; *TBI*, traumatic brain injury. Significant *p*-values < 0.05 are marked bold

A multivariable logistic regression analysis was performed to predict improved outcome 6 months after CP with the following potentially independent variables: age at DC in years (≤ 50/ > 50), sex, side, indication for DC, material, mRS before CP (≤ 3/ > 3), time to CP in days (≤ 149/ > 149), complications (no/yes), and rehabilitation after CP (yes/no). According to multivariable analysis, “mRS ≤ 3” (*p* < 0.001, OR 4.4; 95% CI 2.1–9.3) and “rehabilitation after CP” (*p* < 0.001, OR 4.6; 95% CI 2.2–9.6) were independently associated with an improved outcome after CP (Nagelkerke’s *R*^2^ 19.5%), while the time to CP (*p* = 0.059, OR 1.7; 95% CI 1.0–3.3) was not.

## Discussion

The present study analyzes the effect of the time point of CP on the outcome and complications in a single-center retrospective cohort. Late CP was defined as CP 5 months after DC according to a ROC analysis. Whereas complication rates did not differ between both groups, the neurological outcome subsequently improved more often in patients with an early CP in univariate analysis. This finding could not be validated by multivariate analysis, where a better premorbid (before CP) condition and rehabilitation after CP were independent predictors for outcome improvement after CP.

DC is a common procedure in specific neurological diseases like TBI, IS, SAH, and ICH. The time point for a subsequent CP is controversially discussed. Many clinical institutions perform CP regarding clinical experience at different time points. Functional outcome and complication rate are two important factors for decision-making of the timing of CP. While CP was mainly performed between 3 to 6 months after DC at the authors’ institution, in some cases, CP was performed at a later period. The aim of this study was to evaluate whether complication rate rises and outcome may be worsened when CP was performed at a later period after DC. Therefore, we retrospectively collected data from CP patients in our institution. Our results show that outcome could be improved when patients had a better clinical condition (mRS ≤ 3) before CP and an additional rehabilitation after CP was provided.

Bender et al. already described a better functional outcome when a CP was performed within 86 days after DC. Borger et al. showed that patients benefit more from a CP when the procedure was done 3 or more months after DC [9]. This controversy could be due to the underlying pathologies. Borger et al. analyzed mainly stroke patients, whereas Bender et al. included TBI, IS, SAB. and ICH. Our patient population was as diverse as Bender et al. but showed results like Borger et al. Our results showed that the most favorable outcome was achieved when the CP was performed within 5 months. This could be due to less complications through trauma or stroke-associated immunodeficiency. The benefits of an early CP could be through less atrophy of neurons [3]. Syndromes like “syndrome of the trephined” or “sinking-skin-flap syndrome” are also described in the literature [10, 12, 14]. The existence of these syndromes and their pathogenesis are controversially discussed, but the clinical experience shows that some patients have a physical disability without a morphological correlative in the imaging. Those patients highly benefit from a CP and regain their physical abilities.

It seems not surprising that patients who already achieved a good clinical outcome attended less frequent optional rehabilitation. Nevertheless, rehabilitation after CP is still an independent prognostic factor for improvement of outcome. Bender et al. proclaim concordantly that patients benefit more from an inpatient rehabilitation [10]. However, a better clinical condition before CP also led to an even more favorable outcome after CP. As less patients in a better clinical condition attended rehabilitation after CP, functional outcome 6 months after CP did not differ significantly to those who attended rehabilitation. In univariable analysis, early CP was associated with improved functional outcomes at 6-month follow-up. This association was confounded when rehabilitation after CP was considered. Furthermore, better functional condition before CP (mRS ≤ 3) was associated with more frequent improvement of outcome. While functional condition before CP was different between patients performing additional rehabilitation and not, there was no difference in outcome after 6 months anymore. We therefore conclude that additional rehabilitation after CP is indispensable.

Another controversially discussed topic is the complication rate. Some authors proclaim that an early CP is associated with more complications [3, 6, 9]. Other authors like Bender et al. describe that there is no significant increase of complications when CP is performed early. A prospective multicenter cohort study with more than 500 patients confirmed no differences in complication rates with respect to the interval between DC and CP [15]. Our results also show that there is no significant increase or decrease of complications when a CP is done early (within 5 months after DC) or late. This discrepancy could be due to the underlying pathologies for the DC and its specific molecular changes. TBI patients suffer from a condition that affects the neurometabolites [16, 17]. The regeneration can take up to 3 to 5 months [16, 17]. Trauma-caused skin lesions can also generate secondary infection or can be contaminated. This could be the reason why more complications like infection occur when an early CP is done in trauma patients. IS patients suffer from a stroke-induced immunosuppression [9]. This could be another reason for more complications after an early CP. Borger et al. could show that IS patients suffer more from wound healing disturbances [9]. Schuss et al. also showed that a CP done within 2 months after DC has more complications like wound healing disturbances and infection [6]. In accordance with these data, no CP was performed within 2 months after DC at the authors’ institution. Therefore, the complication rate may show no significant differences in this cohort. In some cases, the brain was swelling intraoperative which is why the operation was postponed. This could be due to the described changes in neurometabolites and the unfinished regeneration process of the brain. Indeed, Bender et al. describe non-significant complications during an early CP, but they also showed that the patients with an early CP had more hydrocephalus. In our study, the early time point for a CP was within 5 months; the other authors classified 2 to 3 months as early time point and > 3 months as late. As a consequence, the listed complications in the other studies for early or late CP are summarized in our early group. Therefore, we can say that we have similar complications, but we cannot discriminate if we have more complications in the early group than the other early groups of other studies. However, we could show that a late CP (after 5 months) has no significant changes in the complication rates.

This study has some limitations. First, the retrospective design of the study. Second, the time point of CP may be subject to selection bias, although this was mitigated by institutional guidelines. This extends to patient factors, such as active infections at the scheduled time point for CP delaying the intervention or readmission being delayed according to the patients’ and their relatives’ wishes. Further external factors were less prone to introduce selection bias, such as the impossibility of offering elective surgeries during some phases of the COVID-19 pandemic. Another limitation may be that patients achieving a good outcome may insist on earlier CP than patients or their next of kin who are still in a bad clinical condition. A prolonged observational interval may show more frequently an improvement of outcome. Therefore, the difference of outcome at the time point of CP and 6 months after was analyzed, not the outcome itself. Lastly, the study cohort consisted of heterogeneous diseases. We excluded some underlying diseases to comply with the most frequent causes for DC that were analyzed in other studies.

## Conclusions

Late cranioplasty is a safe procedure as there were no statistically significant differences in the rate of complications between early and late cranioplasty. The outcome after cranioplasty was improved in patients with better neurological condition before cranioplasty and with additional rehabilitation afterwards.

## Data Availability

All data generated or analyzed during this study are included in this published article.
